# Scientific Items

**Published:** 1879-06-20

**Authors:** 


### SCIENTIFIC ITEMS.

**The Camera Obscura.**—One of the wonders of New York is Central Park, and the wonder of Central Park is the camera obscura "Octagonal." It is a little eight-sided building, surmounted by a small turret furnished with a movable lens. Directly under the turret is a table about five feet in diameter, with a highly polished surface, and on it the scenery of the park and surrounding country is reflected from the lens with marvelous distinctness. Even the country on Long Island, across the East river, is plainly visible, as if one were looking at it through a telescope.

The room will accommodate about a dozen people at a time. It is darkened and the only light admitted enters through the lens and is thrown on the table. It is a most marvelous sight. One may see his friends in any part of the park. The slightest ripple on the lake, or the movement of the leaves on distant trees, is vividly shown on the table, and all the varied and beautiful colors are preserved in the moving miniature picture. Detectives repair to the "Octagonal" when suspected parties are supposed to be in the park. A man or woman walking half a mile away cannot escape the eye of science. Among the many objects to be seen are, Masonic Temple, the Catholtc Cathedral, the churches of upper New York and the Palisades of the Hudson, each appearing in turn as the turret revolves. When one wishes to examine a particular portion of the park, the superintendent gives the machine a turn, and the spot appears on the table, while the bewildered spectator thinks of Aladdin and his lamp, and wonders why, if the days of enchantment have really come, the magician does not transform the dingy room into a palace worthy of such splendid scenes.

**Rapidity of Thought in Dreaming**—A very remarkable circumstance, and an important point of analogy, is to be found in the extreme rapidity with which the mental operations are performed, or rather with which the material changes on which the ideas depend are excited in hemispherical ganglia. It would appear as if a whole series of acts, that would really occupy a long lapse of time, pass ideally through the mind in one instant. We have in dreams no true perception of the lapse of time—a strange property of mind! for if such be also its property when entered into the eternal disembodied state, time will appear to us eternity. The relations of space as well as time are also annihilated, so that almost while an eternity is compressed into a moment, infinite space is traversed more swiftly than by real thought. There are numerous illustrations of this on record. A gentleman dreamed that he enlisted as a soldier, joined his regiment, deserted, was apprehended, carried back, tried, condemned to be shot, and at last led out for execution. After the usual preparations, a gun was fired; he awoke with the report, and found that a noise in the adjoining room had at the same moment produced the dream and awakened him. A friend of Dr. Abercrombie dreamed he crossed the Atlantic and spent a fortnight in America. In embarking, on his return, he fell into the sea, and awaking in the fright found that he had not been in bed ten minutes.

**A New Theory of Electricity.**—Mr. J, C. Moore, of Cincinnati, presented in quite an entertaining lecture before the Academy of Science at its rooms on Mitchell street, what is known as the Chambers' method of protection against lightning. The apparatus employed consists of a rod of galvanized iron bent so as to form three sides of a parallelogram, which is set upright upon the roof of the building with the points vertical and the horizontal section insulated, a foot or more from the ridge of the roof. There is, as will be perceived, no ground connection whatever, the plan, in this particular, differing from all others which have preceded it. Mr. Moore insisted that such apparatus by instantly diffusing the negative electricity which accumulates on the top of the building tends to prevent a stroke of lightning, and that should a charge of electricity be received from an overhanging cloud it would be harmlessly dispersed into the atmostphere from the points upon the rod. The theory urged was fully sustained by a series of interesting experiments, and there appeared no reason to question its correctness as much as it contradicts our preconceived opinions on the subject. The lecturer also exhibited a new form of insulator so made as certainly to keep dry at the place of contact with the building, the advantage of which is unquestionable.—*Ex.*

**Gallium.**—Lecoq de Boisbraudan and E. Jungfleisch have published in *Comptes Rendus* some more notes on their examination of the properties of this newly-discovered and curious element. The metal crystallizes in octahedra with the summits of the pyramid cut off by a plane, in some cases truncated so as to give the crystals a tabular form. The metal is hard and only to a small degree malleable, although thin plates may be bent backwards and forwards many times without breaking. A ray of light which has been reflected several times from bright surfaces of the metal acquires a fine bluish-green color.—*Journal of Chemistry.*

**The New Metal Philippium.**—Mr. W. G. Brown, of the East Tennessee University, writes to the *Chemical News* that while analyzing *sipylite*, a year or more ago, he observed with the spectroscope certain lines, one at least of which appears to belong to the new metal phillippium, and indicates the presence of that element in sipylite.—*Journal of Chemistry.*

**Gossamers.**—Gossamers, noticed upon meadows and prairies in pleasant summer and autumn weather, are spun by diminutive spiders. In some cases they float in the air and in others they are attached to grass-tufts. They prove serviceable in ensnaring the spider's prey or collecting dew for the little creature to drink.

**Longevity and Civilization.**—Ethnologists and statisticians agree that life is of longest duration in countries were people are most civilized. A great number of people are born in uncivilized communities, but fewer reach maturity than among the civilized.

**Alpha.**—The fixed star nearest to the earth is Alpha, in the constellation Centaur; yet it is 86,000,000 times as far from us as the moon. It performs its revolutions once in seventy-eight years, and is a star of the first magnitude.

